# Antibacterial effect and mechanism of anthocyanin from *Lycium ruthenicum Murr*

**DOI:** 10.3389/fmicb.2022.974602

**Published:** 2022-08-18

**Authors:** Yuhe Dong, Chunmiao Yang, Wenting Zhong, Yan Shu, Yongze Zhang, Dongsheng Yang

**Affiliations:** ^1^College of Pharmacy and Food Science, Zhuhai College of Science and Technology, Zhuhai, China; ^2^School of Life Sciences, Jilin University, Changchun, China; ^3^Guangdong-Macao TCM Science and Technology Industrial Park Development Co. Ltd., Zhuhai, China

**Keywords:** *Lycium ruthenicum Murr*, anthocyanin, *S. aureus*, antibacterial mechanism, biofilm

## Abstract

The inhibitory effects of the anthocyanin obtained from *Lycium ruthenicum Murr* were tested against several food-borne pathogens were evaluated, such as *Staphylococcus aureus* (*S. aureus*), *Escherichia coli* (*E. coli)*, *Aspergillus niger* and *Penicillium sp.* In general, anthocyanin had different antibacterial effect on different bacteria, and the best antibacterial effect on *S. aureus*, with minimal inhibitory concentration (MIC) of 3.125 mg/mL. Anthocyanin increased the surface hydrophobicity of *S. aureus*, discharged the intracellular K^+^, and reduced the total soluble protein, affecting protein synthesis. Fluorescent inverted microscope and flow cytometry (FCM) found a significant increase in fluorescence intensity and lethality relative to the control group, and the dead P3 region to 77.21%. The above suggested a correlation between the antibacterial mechanism of anthocyanin and cell membrane permeability integrity. Biofilm formation was evaluated by the crystal violet assay (CV), silver staining method and methyl thiazolyl tetrazolium (MTT). Scanning electron microscopy (SEM) showed that anthocyanins could change the structure of biofilm.

## Introduction

*Lycium ruthenicum Murr.* (LR), belonging to the Lycium genus of the Solanaceae family, is distributed in northern Shanxi, Ningxia, Gansu, Qinghai, Xinjiang, and Tibet. It has been widely used in Tibetan medicine and Uyghur medicine. According to the Tibetan medicine classics such as “The Four Medical Tantras” (Yu and Yuan, 1983) and “Jing Zhu Materia Medica” (Timar, 1986), LR is called “Pang Ma,” and is used in the treatment of heart heat disease, heart disease, irregular menstruation, menopause. According to the “Uygur Medicine,” the fruit and root bark of LR are commonly used in the treatment of urethral stones, scabies, gingival bleeding and other diseases, and for building up the body and lower blood pressure among the common people (Gan et al., 1995). LR is sweet in taste, neutral in nature, rich in a variety of nutrients, such as protein, *Lycium barbarum* polysaccharides (LBP), polyphenols, anthocyanin, flavonoids, and trace elements (Xia et al., 2015; Dong et al., 2020; Azzejiang et al., 2021).

Natural antibacterial drugs have a long history of medicinal use in China. Searching for higher efficiency and lower toxicity antibacterial substances from natural plants is a current hot research topic. It has been reported that natural antibacterial drugs have good antibacterial effects in *in vitro* bacteriologist experiments (Zhao et al., 2019). Thus, the unique advantages of natural antibacterial drugs in the field of bacteriologist can provide new ways and ideas for clinical treatment of infectious diseases as well as bacteriologist of food and cosmetics. The antibacterial mechanism of natural drugs is mainly divided into two categories: the first is direct antibacterial mechanism, that is, to kill or inhibit the growth and reproduction of bacteria by directly acting on them; the second is indirect antibacterial mechanism, that is, to stimulate the immune response of the organism by improving its immunity to achieve the antibacterial effect (Tian, 2019).

There have now been many studies on direct antibacterial mechanism, which includes inhibiting the generation of energy (Sato et al., 2004), changing the cell membrane permeability and ion channels, inhibiting the enzyme activity in bacteria, enhancing the pharmaceutics function of eutrophic (Zhang et al., 1982), reducing the release of bacterial antitoxins (Chang et al., 2007), promoting the metabolites to play antibacterial effects (Yan et al., 2003), inhibiting the synthesis of proteins, inhibiting the biofilm formation of bacteria (Gan, 2007). Nevertheless, further research on the antibacterial mechanism of TCM will help to further explore the antibacterial mechanism and its deep application of TCM.

To form biofilm, bacteria produce extra cellular polymeric substances (EPS) (Flemming and Wingender, 2010) composed of proteins, carbohydrates, extra cellular DNA, and lipids that provide mechanical stability to biofilm, which wraps bacteria in a viscous matrix promoting their survival to deal with extreme environment (Alpkvist et al., 2006). The biofilm has four stages of developmental processes: initial colonization, aggregation, maturation and diffusion. *S. aureus*, with such a similar biofilm structure (Davies, 2003; Hall-Stoodley and Stoodley, 2009; Bhattacharya et al., 2015), is often found during food processing. Due to the presence of biofilm, *S. aureus* is strongly resistant to disinfectants. So it not only enhances the carcinogenicity of pathogenic bacteria, but also increases the risk of microbial contamination in food safety. There have been many domestic and foreign studies of the effect of anticyclonic on antibacterial effect, but little research on its ability to clear biofilm. Anthocyanin, a safe and non-toxic natural plant pigment, has a variety of physiological functions. With people’s deeper understanding of natural plant pigments, anticyclonic with unique biological activity has broad development prospects. There are abundant anthocyanin in LR, which, however, are rarely reported in their bacteria inhibition and antibacterial mechanism. This paper aims to expand their application path and provide a theoretical basis for their further development into drugs, food and cosmetics.

## Materials and methods

### Strains, materials, and reagents

All bacterial strains were from the GuangDong Microbial Culture Collection Center (GDMCC).

K^+^ Assay Kit (Nanjing Jiancheng Technology Co., Ltd.), BCA Protein Assay Kit, SDA-PAGE kit (Beijing Dingsheng Biotechnology Co., Ltd.), Nutrient Broth (NB), Nutrient Agar (NA), Mueller hinton Broth (MH), Potato Dextrose Water (PDW), Trypticase Soy Broth (TSB), LB, 0.5% 2, 3, 5-Triphenyltetrazolium chloride (TTC) (Guangdong Huankai Microbial Sci. &Tech. Co., Ltd.); Hexadecane (Xilong Scientific Co., Ltd.); PI/Nase staining solution (Beijing Solarbio Science & Technology Co., Ltd.).

### Instruments and equipment

Electronic Analytical Balance (FA2204B, Shanghai Instrument and Meter Co., Ltd.); Refrigerated Centrifuge (1580R, GeneHarbor HK Limited); Pressure Steam Sterilizer (YXQ-LS-75SII, Shanghai Boxun Medical Biological Instrument Corp.); Reciprocating Constant Temperature Shaking Water Bath (ZHWY-110 × 50, Changzhou NuoJi Instrument Co., Ltd.); Wheat Turbidity Comparator (TA-2XJB, Beijing Tian’an United Technologies Co., Ltd.); ELISAreader (Epoch, Bio Tek Instruments inc., United States); OM (FA2204B, Shanghai Instrument and Meter Co., Ltd.); Fluorescent Inverted Microscope (LW300LFT, Shanghai Cewei Photoelectric Technology Co., Ltd.); flow cytometry (FCM) [CyoFLEX S, Beckman Coulter Commercial Enterprise (China) Co., Ltd.]; Scanning electron microscopy (SEM) (MIRA TESCA).

### Experimental methods

#### Strain activation and culture

The stored bacteria were subcultured twice to ensure freshness. Then, they were cultured in an incubator at 37°C for antibacterial experiments.

#### Preparation of anthocyanin extract from LR

In our preliminary study, we performed UV full wavelength scan of the crude extract and found that it has a characteristic peak at 517 nm and has the property of turning red when exposed to acid and blue when exposed to base. LR were accurately weighed, crushed, screened, and then extracted under conditions of 1% HCL-70% ethanol, ultrasonic power 216 W, microwave power 89 W, time 26 min, the ratio of liquid to material 17:1 mL/g to obtain the crude anthocyanin extract, some of which was purified with AB-8 resin to obtain the purified anthocyanin. Both the crude anthocyanin extract and purified anthocyanin were concentrated by reducing pressure and freeze-dried to obtain their dry powder, which was then stored at −20°C.

#### Minimal inhibitory concentration

The crude anthocyanin extract and purified anthocyanin were dissolved in MH or PDW, and added to 96-well. After 100 μL of bacterial suspension (10^5^ CFU/mL) was added to the first well of each row and mixed, 100 μL of the resulting mixture was absorbed and added to the second well of each row by double dilution method. Two other wells were taken out, one was added with levofloxacin as a positive control and the other not added with bacterial suspension as a negative control. For *S. aureus* and *E. coil*, each well was added with 5 μL of 0.5% (TTC) solution, mixed, and cultured at 37°C (Ma and Kuai, 1996). And the lowest concentration of anthocyanins to the fungus can be observed by eye as the concentration at which spores first appear.

#### Effect of anthocyanin on the growth of *Staphylococcus aureus*

Activated *S. aureus* was taken and placed in the LB, and the concentration of the resulting *S. aureus* suspension was adjusted to 10^5^ CFU/mL. Then, different concentrations of anthocyanin were added to three *S. aureus* suspensions until their final mass concentration reached 1/2 MIC, MIC and 2 MIC, respectively, with the *S. aureus* suspension without anthocyanin as the control group. Four separate centrifuge tubes containing *S. aureus* suspension treated with different concentrations of anthocyanin were then placed at 37°C for 120 r/min shaking culture. After 0, 0.5, 3, 6, 10, and 24 h of culture, respectively, appropriate *S. aureus* suspension was appropriately diluted and evenly coated on the agar medium; colonies were counted after 16 h of culture in a 37°C incubator (Zhang, 2016).

#### Effect of anthocyanin on the surface hydrophobicity of *Staphylococcus aureus* cells

The microbial adhesion to hydrocarbons (MATH) (Pelletier et al., 1997) was adopted. A certain volume of *S. aureus* suspension in logarithmic growth phase was taken and centrifuged, and then washed three times with Phosphate buffer saline (PBS) and resuspended to OD_600_ nm = 0.5. A total of 4 mL of the above *S. aureus* suspension was taken and put into three centrifuge tubes. Then, different concentrations of anthocyanin were added to two *S. aureus* suspensions until their final mass concentration reached 1/2 MIC and MIC, respectively, with the last *S. aureus* suspension without anthocyanin as the control group. An equal volume of hexadane was subsequently added to the three *S. aureus* suspensions. After being fully mixed, the three *S. aureus* suspensions were incubated for 10–15 min at room temperature to ensure complete separation between water and hexadane without *S. aureus* settling in solution. The aqueous phase was absorbed and the OD at 600 nm was measured. The adsorption rate was calculated according to the following formula:


(1) OD(%)=(OD0-OD1)/OD0×100%

Where OD0 and OD1 were the absorbance before and after extraction with hexadecane, respectively.

#### Effect of anthocyanin on the K^+^ content of *Staphylococcus aureus*

The *S. aureus* suspension in logarithmic growth phase was taken and centrifuged at 4°C, 4,000 rpm for 10 min, and then the *S. aureus* was collected. The above *S. aureus* suspension was then washed three times with PBS and resuspended, and the concentration was adjusted to 10^8^ CFU/mL. The PBS suspension was the control group, and the PBS suspension added with anthocyanin (MIC) was the experimental group. Both of them were placed in a constant temperature shaker for determination. The samples were drawn every 30 min, centrifuged at 4°C, 4,000 rpm for 10 min. According to the operation method of the K^+^ Assay Kit, the supernatant was collected to detect the K^+^ content in the suspension (Huang, 2021). Parallel determination for three times.

#### Effect of anthocyanin on the intracellular protein and synthesis of *Staphylococcus aureus*

The *S. aureus* suspension in logarithmic growth phase was taken and centrifuged at 4°C, and then washed three times then the bacteria were diluted in sterile PBS (10^8^ CFU/mL). Then, different concentrations of anthocyanin were added to two suspensions until their final mass concentration reached MIC and 2 MIC, respectively, the suspension without anthocyanin as the control group. They were incubated at 37°C for 3, 6, and 9 h. Then 1 mL of suspension was taken and centrifuged at 4°C, 4,000 rpm for 10 min. The precipitated *S. aureus* was collected and resuspended with 1 ml of PBS, and then disrupted by ultrasonic in an ice bath for 10 min. The absorbance at 526 nm were determined and recorded according to the BCA Protein Assay Kit. Then, the above *S. aureus* was collected by centrifugation at 4°C, and suspended in 40 μL of sterile double distilled water. It was then added with the same volume of 2 Loading Buffer and put in boiling water to boil for 5 min. And then it was centrifuged at 8,000 rpm for 5 min after cooling. The resulting supernatant was used for SDS-PAGE, after which the protein gel was stained in Coomassie brilliant blue for 15 min and then decolored, until the protein bands were clearly visible (Bai, 2019).

#### Effect of anthocyanin on the lethality of *Staphylococcus aureus*

The *S. aureus* suspensions with a concentration of 10^7^ CFU/mL were prepared. Then, different concentrations of anthocyanin were added to two suspensions until their final mass concentration reached MIC and 2 MIC, respectively, with the last suspension without anthocyanin as the control group. All of them were shaken up and cultured at 37°C overnight. Then, 1 mL of suspension was taken and washed three times with PBS. Then it was centrifuged and the supernatant was removed. After being added with 500 μL of PI/RNase staining solution, it was stained away from light for 15 min. Then, it was centrifuged, washed twice with sterile double distilled water, and resuspended with 500 μL of sterile double distilled water. Finally, the lethality was observed by fluorescence inverted microscope and rapidly detected by FCM (Gottfredsson et al., 1998).

#### Effect of anthocyanin on the biofilm formation of *Staphylococcus aureus* by silver staining method

The sterile cover slides were soaked in 75% ethanol solution and sonicated for 30 min to remove the grease on their surface, and then rinsed with sterilized water and autoclaved at 121°C for later use. A total of 5 mL of anthocyanin (MIC and 2MIC) dissolved in TSB was added to each well of a 12-well cell culture plate, with the well without anthocyanin as the control group, followed by 500 μL of *S. aureus* suspension (10^8^ CFU/mL). Several sterile cover slides were put on each well for thermostatic static culture at 37°C for 2 day. The cover slides were observed by silver staining method (Xu, 2017).

#### Biofilm biomass assay (modified crystal violet assay)

The *S. aureus* was inoculated in TSB for culture overnight. After a 200-fold dilution, 100 μL of the resulting *S. aureus* suspension was added to each well of a 96-well cell culture plate. Then, different concentrations of anthocyanin were added to suspensions until their final mass concentration reached 1/4 MIC∼4 MIC, respectively, the suspension without anthocyanin as the control. All the solutions were cultured overnight at 37°C. After the bacterioplankton was washed away with PBS, Bouin’s fluid was added for 1 h. The solutions were washed three times with PBS, and then stained with 0.1% crystal violet for 30 min, and the loose color was washed away with sterile water. After the biofilm dried, 200 μL of ethanol solution: acetone = 80:20 was added to each well. The absorbance was measured at 570 nm (Djordjevic et al., 2002; Tetz et al., 2009).

#### Effect of anthocyanin on the biofilm metabolism of *Staphylococcus aureus* by methyl thiazolyl tetrazolium staining method

After washing three times with PBS, 250 μL of 0.5 mg/mL methyl thiazolyl tetrazolium (MTT) solution was added to each well of a 96-well cell culture plate. After the resulting solutions were incubated at 37°C for 3 h, the MTT solution was removed, and they were dissolved with 250 μL of DMSO. The absorbance was measured at 570 nm (Schillaci et al., 2008).

#### Scanning electron microscope analysis

The suspensions (10^7^ CFU/mL) were added 1 and 2 MIC of anthocyanin, respectively; the control was conducted without anthocyanin. After cultured at 37°C for 16 h, a large amount of suspension was centrifuged at 4°C, 8,000 r/min for 5 min, and the supernatant was removed. After the suspension was added with 2.5% glutaraldehyde solution 40 times the volume of suspension was added and fixed at 4°C for 6 h, it was washed three times with PBS. The *S. aureus* was dehydrated with 30, 50, 70, 70, 80, 90% ethanol solution, respectively for approximately 15 min, and then dehydrated twice with 100% ethanol solution. The *S. aureus* was then placed in a mixture of alcohol-tert-butyl alcohol (1:1) and pure tert-butyl alcohol, respectively, for 15 min, and the mixed suspension of *S. aureus* and tert-butyl alcohol was absorbed and dropped on the cover slides. The cover slides containing the sample was put in a −20°C refrigerator for 30 min, and then removed for overnight to let the tert-butyl alcohol evaporate. After the samples fully dried, the cover slides were glued to the sample stage with a conductive tape, gold-plated, and observed by SEM (Saritha et al., 2015).

#### Statistical analysis

Each experiment was performed in triplicate and repeated three times. All data were presented as mean ± standard deviation (SD). Subsequently, the results were analyzed in Origin 2018 statistical software by using One-Way ANOVA method. *P* < 0.05 was considered as significant.

## Results and discussion

### Antibacterial effect of anthocyanin

The principle for TTC colorimetric method to determine MIC: colorless TTC reacts with dehydrogenase in living bacteria to generate red but water-insoluble TPF, which is stable and cannot be automatically oxidized by oxygen in the air; TTC will not be reduced if there are no or small living bacteria, so the solution remains colorless. So TTC can serve as a color-developing agent to determine bacteria survival (Zhang et al., 2009). Evaluation of antibacterial activity of purified anthocyanin is shown in Table 1. As can be seen in Table 1, the purified anthocyanin had some antibacterial activity against the test strains at the preset concentrations. The MIC of purified anthocyanin for *Aspergillus niger*, *Penicillium* sp., *E. coli*, and *S. aureus* was 25, 12.5 mg/mL and 6.25 and 3.125 mg/m, respectively. The results showed that LR had good antibacterial effects on *S. aureus*. This is consistent with the results (Xiang et al., 2016), but the antibacterial effect of LR is better than that of crude ethanol solution extract.

**TABLE 1:** Evaluation of antibacterial activity of purified anthocyanin.

Type of bacteria	Concentration (mg/mL)							MIC
	50	25	12.50	6.25	3.125	1.563	0.75	
*S. aureus*	−	−	−	−	−	+	+	3.125
*E. coli*	−	−	−	−	+	+	+	6.25
*Aspergillus niger*	−	−	+	+	+	+	+	25.0
*Penicillium sp.*	−	−	−	+	+	+	+	12.5

### Effect of anthocyanin on the growth of *Staphylococcus aureus*

**FIGURE 1:**
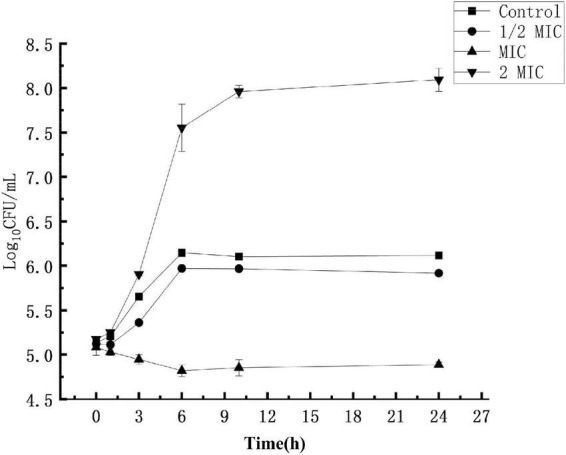
Effect of anthocyanin on *Staphylococcus aureus* growth.

The experimental results are shown in Figure 1. In the control group where the *S. aureus* suspension was not treated with anthocyanin, the *S. aureus* colonies increased exponentially, and their growth was not inhibited. After 24 h, the number of *S. aureus* colonies grew from 5.17-log10 CFU/mL to 8.09-log10 CFU/mL, up by 3 logarithm values. In the experimental groups, the *S. aureus* colonies increased exponentially, and their growth was not significantly inhibited, but slightly inhibited compared to the control group. After 24 h, the number of *S. aureus* colonies grew to 6.11-log10 CFU/mL and 5.91-log10 CFU/mL, respectively. When the *S. aureus* suspension was treated with anthocyanin (2 MIC), the growth state of *S. aureus* colonies showed a downward trend, and their growth began to be largely inhibited. After 24 h, the number of *S. aureus* colonies was reduced from 5.08-log10 CFU/mL to 4.88-log10 CFU/mL. This showed that the growth of *S. aureus* was increasingly inhibited with the increasing concentration of anthocyanin.

### Effect of anthocyanin on the surface hydrophobicity of *Staphylococcus aureus*

**FIGURE 2:**
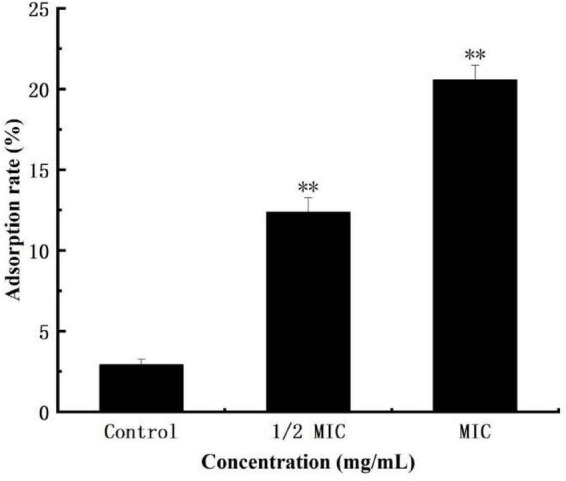
Effect of anthocyanin on the surface hydrophobicity of *Staphylococcus aureus* cells control. ^**^P < 0.01.

The surface hydrophobicity of bacterial cells is an important factor in bacterial growth and controlling bacterial adhesion. The higher the surface hydrophobicity, the easier the sinking of bacteria, and the more effect on the growth of bacteria (Hou et al., 2007; Azimi et al., 2021). Figure 2 shows that treatment of *S. aureus* suspensions with different concentrations of anthocyanin (respective 1/2 MIC and MIC) caused confusion and damage to *S. aureus* cell membrane, and increase in the surface hydrophobicity of *S. aureus* cells. And the surface hydrophobicity increased with increasing concentration of anthocyanin.

### Effect of anthocyanin on the extracellular K^+^ of *Staphylococcus aureus*

The cell membrane of bacteria is easy to be damaged after being stimulated, causing the leakage of intracellular ions such as K^+^ and metabolic disorders. At the same time, some soluble substances in the bacterial cells will be released (Cox et al., 2001; Diao et al., 2014). Small molecule electrolytes preferentially leak than large molecules such as nucleic acids and proteins (Haktanir et al., 2021). Damage to the cell membrane of bacteria disrupts the K^+^ channel and affects the Na^+^-K^+^-ATP enzyme activity, which will lead to the failure to complete the cell membrane transport-based metabolism and induce premature programmed cell death.

**FIGURE 3:**
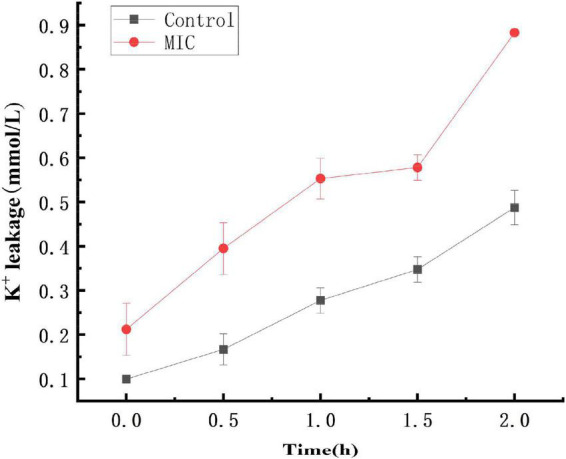
Effect of anthocyanin on the extracellular K^+^ of *Staphylococcus aureus*.

As shown in Figure 3, within 0–2 h, the extracellular K^+^ concentration in both the control and anthocyanin (MIC)-treated groups increased, with the former lower than the latter. The K^+^ concentration increased from 0.09L to 0.48 mmol/L in the control group, and 0.21 to 0.88 mmol/L in the anthocyanin (MIC)-treated group. The significant upward trend of K^+^ concentration indicated that anthocyanin can change the *S. aureus* cell membrane permeability, resulting in massive K^+^ leakage to outside the *S. aureus* cells.

### Effect of anthocyanin on the intracellular protein and synthesis of *Staphylococcus aureus*

**FIGURE 4:**
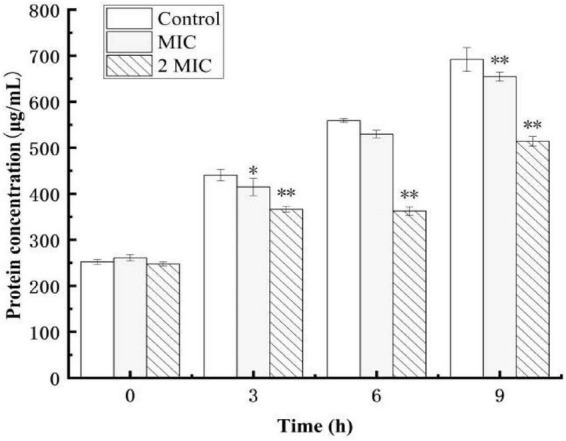
Effect of anthocyanin on the intracellular soluble proteins of *Staphylococcus aureus*. *P < 0.05, ^**^P < 0.01

**FIGURE 5:**
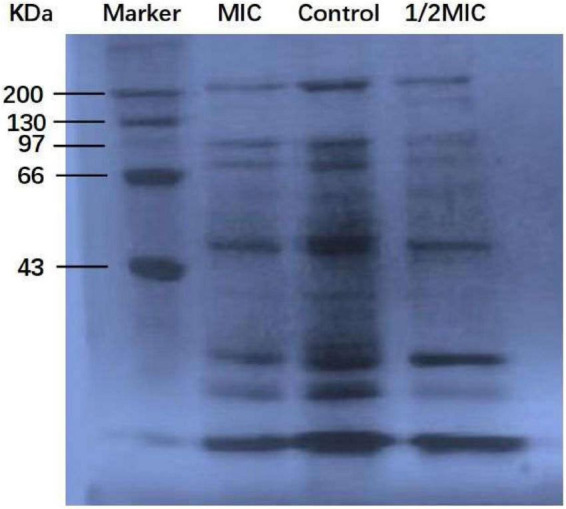
SDS-PAGE images of protein.

Protein, the material basis for bacterial life activity, plays an important role in the physiological metabolism of bacteria. When the bacterial protein synthesis capacity is inhibited, the normal physiological metabolism of bacteria will be affected, thereby inhibiting bacterial growth. The test results of the BCA Protein Assay Kit are shown in Figure 4. As shown in Figure 4, after 9 h of culture, the total soluble protein increased from 252 μg/mL to 692 μg/mL in the control group, but decreased by 5.5 and 25.7%, respectively, in the anthocyanin (respective MIC and 2MIC)-treated groups, with significant differences (*P* < 0.05). The results showed that anthocyanin reduced the content of soluble protein in bacteria. This is consistent with the SDS-PAGE. Figure 5 shows that the *S. aureus* protein bands in the anthocyanin (respective MIC and 2MIC)-treated groups significantly decreased when compared to the control group, indicating that anthocyanin enhanced protein leakage.

### Effect of anthocyanin on the lethality of *Staphylococcus aureus*

**FIGURE 6:**
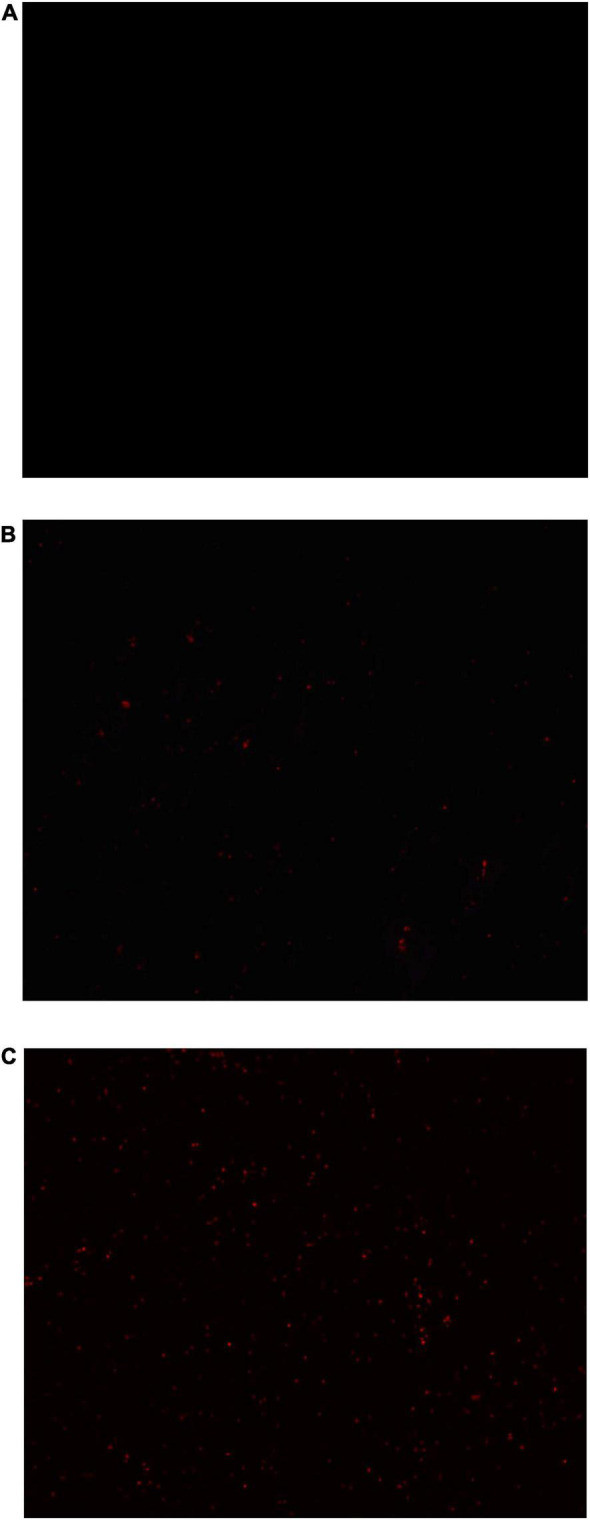
Images of *Staphylococcus aureus* treated with anthocyanin (respective MIC and 2 MIC) by fluorescent inverted microscope. **(A)** Is the control group; **(B)** is the anthocyanin (MIC)-treated group; **(C)** is the anthocyanin (2 MIC)-treated group.

The Propidium iodide (PI) staining results of *S. aureus* suspensions treated with anthocyanin (respective MIC and 2 MIC) are shown in Figure 6. For normal undamaged bacteria, PI cannot penetrate their membrane to bind to their DNA, so fluorescence will not be observed; for damaged bacteria, PI can penetrate their membrane to bind to their DNA, so fluorescence will be observed. So the observed fluorescence can be used to reflect the bacterial membrane damage (Chakotiya et al., 2017).

Figure 6A is the control group not treated with anthocyanin, where no fluorescence was observed, indicating that no *S. aureus* membrane was damaged. Figure 6B is the anthocyanin (MIC)-treated group, where a small amount of fluorescence was observed, indicating that the normal growth of *S. aureus* was affected due to anthocyanin, and some *S. aureus* died. Figure 6C is the anthocyanin (2 MIC)-treated group, where a large amount of fluorescence was observed, indicating that anthocyanin changed the membrane permeability of *S. aureus* and caused its death, so PI penetrated its membrane to bind to its DNA, exciting fluorescence. This showed that anthocyanin may affect the normal growth and metabolism of *S. aureus* by changing its membrane permeability. And with increasing concentration of anthocyanin, the fluorescence intensity increased, and the number of *S. aureus* with damaged membrane increased.

**FIGURE 7:**
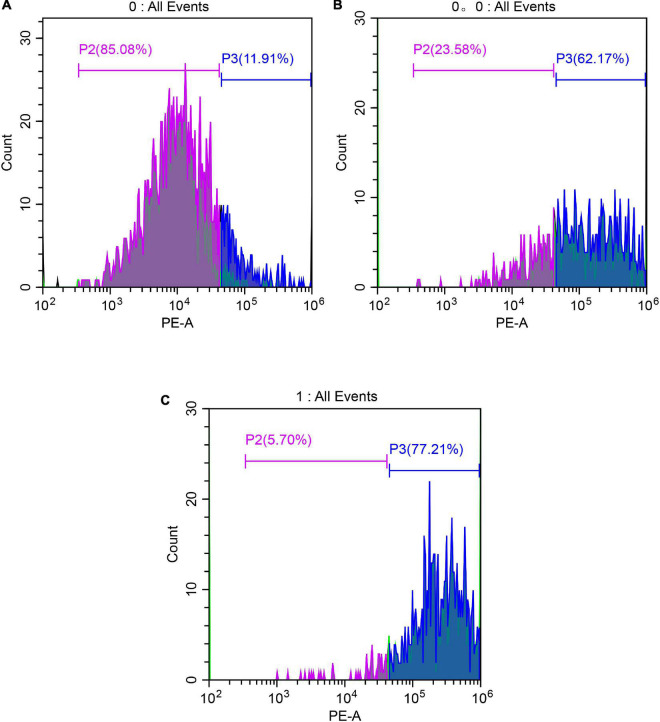
Images of *Staphylococcus aureus* treated with anthocyanin (respective MIC and 2 MIC) by FCM. **(A)** Is the control group; **(B)** is the anthocyanin (MIC)-treated group; **(C)** is the anthocyanin (2 MIC)-treated group.

The lethality of *S. aureus* was detected by FCM (Figure 7). As shown in Figure 7, in the control group (A), the fluorescence was in the left side, with weak fluorescence intensity and only 11.91% in the P3 region, indicating that no *S. aureus* membrane was damaged; in the anthocyanin (MIC)-treated group, the fluorescence moved to the right, with significantly increased fluorescence intensity and 62.17% of the P3 region, indicating that more than half of *S. aureus* was damaged and its membrane permeability was changed; in the anthocyanin (2 MIC)-treated group, the fluorescence increased, with significantly increased fluorescence intensity and 77.21% of the P3 region, indicating that with increasing concentration of anthocyanin, the fluorescence intensity increased, and the number of *S. aureus* with damaged membrane increased, and more PI penetrated the membrane to bind to DNA, causing more *S. aureus* deaths.

### Effect of anthocyanin on the biofilm formation of *Staphylococcus aureus* by silver staining method

**FIGURE 8:**
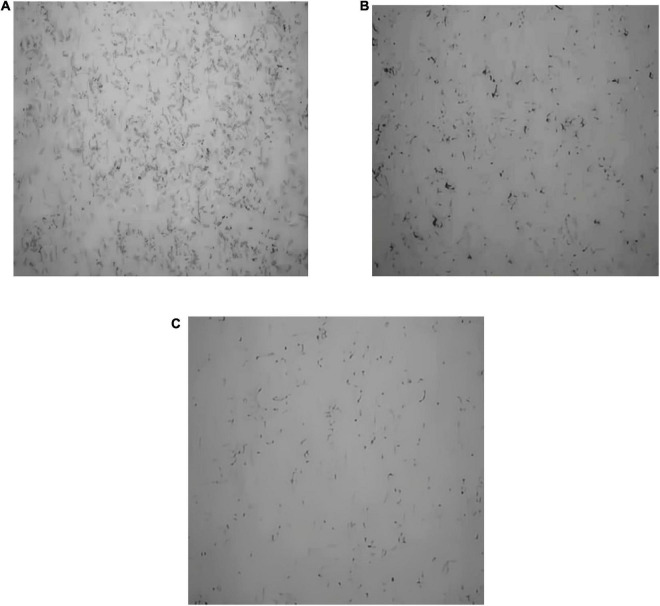
Effect of anthocyanin on the biofilm formation of *Staphylococcus aureus* by silver staining method. **(A)** Is the control group; **(B)** is the anthocyanin (MIC)-treated group; **(C)** is the anthocyanin (2 MIC)-treated group.

The black clumps are formed from *S. aureus* biofilm after silver staining (Figure 8). The biofilm formed from *S. aureus* after 2 days of culture was stuck to the cover slides. As shown in Figure 8, in the control group, a thick layer of dense biofilm on the cover slide was observed; in the anthocyanin (MIC)-treated group, the black clumps became smaller; in the anthocyanin (2 MIC)-treated group, the black clumps became increasingly smaller and looser, indicating that anthocyanin can basically inhibit the biofilm formation of *S. aureus*.

### Effect of anthocyanin on the biofilm clearance of *Staphylococcus aureus* by semi-quantitative (modified cv method) method

**FIGURE 9:**
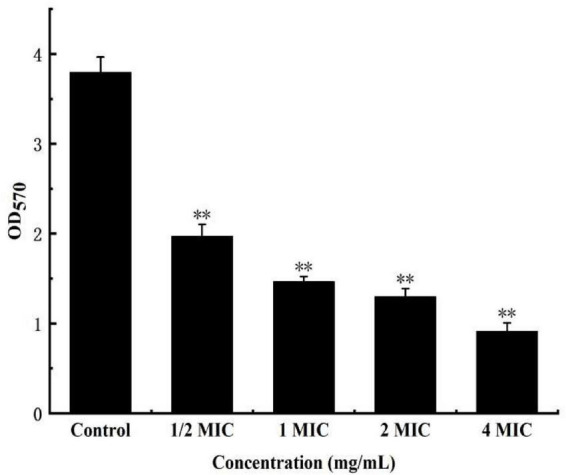
Effect of anthocyanin on the biofilm clearance of *Staphylococcus aureus* by semi-quantitative (modified crystal violet staining method) method. ^**^P < 0.01.

The cv staining method can be used as an indicator of attached biomass on the biofilm. As a more reliable method for quantifying total biomass, it marks living and dead cells as well as extracellular matrix, which allows quantifying total biomass (Kouidhi et al., 2010; Makovcova et al., 2017). As shown in Figure 9, when the concentration of anthocyanin reached 1/2 MIC, the number of biofilms decreased significantly (*P* < 0.01), a onefold decrease compared with the control group; and the number of biofilms decreased more obviously with the increasing concentration of anthocyanin; when the concentration of anthocyanin reached 4 MIC, the number of biofilms was only 1/3 of the control group, indicating that low concentrations of anthocyanin can achieve biofilm clearance.

### Effect of anthocyanin on the biofilm metabolism of *Staphylococcus aureus* by methyl thiazolyl tetrazolium staining method

**FIGURE 10:**
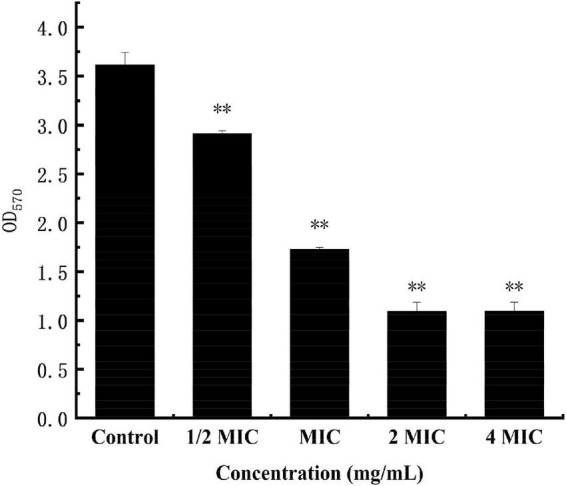
Effect of anthocyanin on the biofilm metabolism of *Staphylococcus aureus* by MTT staining method. ^**^P < 0.01.

The cv staining method can be used as an indicator of attached biomass on the biofilm, but it can’t reveal the metabolism of cells The MTT staining method is to detect cell survival and growth. The succinate dehydrogenase in the mitochondria of living cells can reduce MTT to water-insoluble blue-violet crystal methyl, which can be dissolved by DMSO. However, this is not the case for dead cells. The MTT staining method thus can be used as a respiratory indicator of living cells with metabolic function (Krom et al., 2007). As shown in Figure 10, in the anthocyanin (1/2 MIC)-treated group, the biofilm metabolism of *S. aureus* was significantly inhibited (*P* < 0.01) compared with the control group; and the biofilm metabolism of *S. aureus* was inhibited more obviously with the increasing concentration of anthocyanin.

### Scanning electron microscope analysis

**FIGURE 11:**
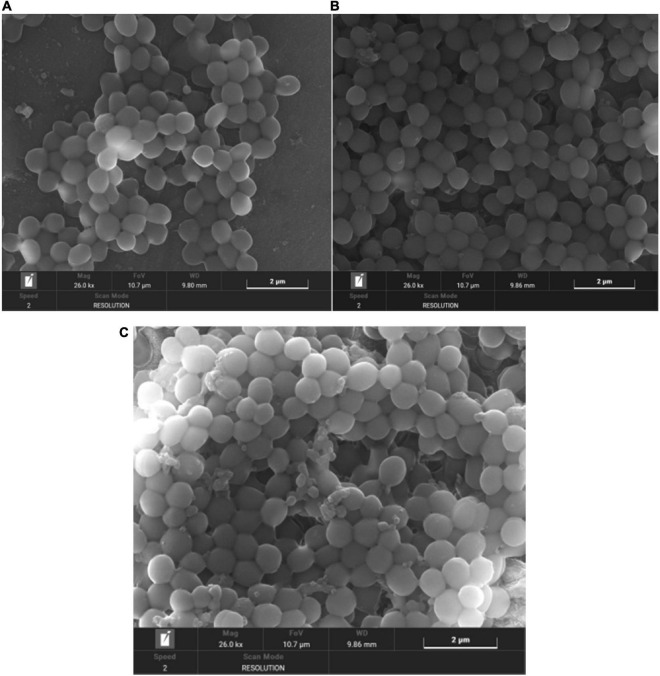
**(A)** SEM (×26000) images of *Staphylococcus aureus* biofilm **(B)** SEM (×26000) images of biofilm of *S. aureus* treated with anthocyanin (MIC) **(C)** SEM (×26000) images of biofilm of *S. aureus* treated with anthocyanin (2 MIC).

Scanning electron microscope can observe microbial biofilms and their detailed structure surface morphology (Makovcova et al., 2017). As shown in Figure 11, in the control group, the *S. aureus* biofilm is a thick, mature and tight three-dimensional structure with smooth surface; in the anthocyanin (MIC)-treated group, the structure was damaged and became loose; in the anthocyanin (2 MIC)-treated group, the *S. aureus* aggregation was more obvious as the scattered free *S. aureus* aggregated to form small clusters. The rupture of *S. aureus* was observed, as some fragments around *S. aureus* and a large amount of content leaked and attached to the surface. Thus, anthocyanin can damage the biofilm structure of *S. aureus*.

## Conclusion

This study aims to explore the antibacterial mechanism of anthocyanin from LR on four foodborne pathogen. It has a good antibacterial effect on bacteria, especially *S. aureus*. The determination of protein, K^+^ leakage and PI staining method showed that anthocyanin caused *S. aureus* cell death by damaging its cell membrane integrity; anthocyanin can effectively achieve *S. aureus* biofilm clearance by inhibiting its biofilm metabolism, which was supported by the SEM observations. Whether the antibacterial effect of anthocyanin was played by one, or by multiple components as it is a mixture was not identified in this study. So further studies are needed to demonstrate the practical use of anthocyanin as a natural antibacterial agent in food to fully understand the mechanisms of its action, including antibacterial effects on other foodborne pathogens, transmission electron microscope investigations, and precise sites of inhibition at the molecular level. And the stability of anthocyanins is poor. In the future, anthocyanins can be encapsulated to prepare more stable and long-release anthocyanin inclusion compounds, and pH-responsive antibacterial materials can be prepared using their antibacterial and antioxidant effects as well as the difference in color and structure under different pH conditions.

## Data availability statement

The original contributions presented in the study are included in the article/supplementary material, further inquiries can be directed to the corresponding author.

## Author contributions

YD and DY: conceptualization, methodology, and data curation. YD: writing original draft. WZ, YS, and YZ: preparation. WZ and CY: software, validation, and writing- reviewing and editing. DY: supervision. All authors discussed the results, read, and revised the manuscript and approved the submitted version.
